# Supplementary material for: The interactome of intact mitochondria by cross-linking mass spectrometry provides evidence for coexisting respiratory supercomplexes
Source: Mol Cell Proteomics. 2017 Dec 8;17(2):216–32. doi: 10.1074/mcp.RA117.000470 (PMC5795388; doi:10.1074/mcp.RA117.000470)
Supplement: Supplemental Data [file supp_RA117.000470_133922_0_supp_23978_fzffwf.zip › spectra_annotation/mito_spectra_annotation/105-1-7-1-13-1.pdf]

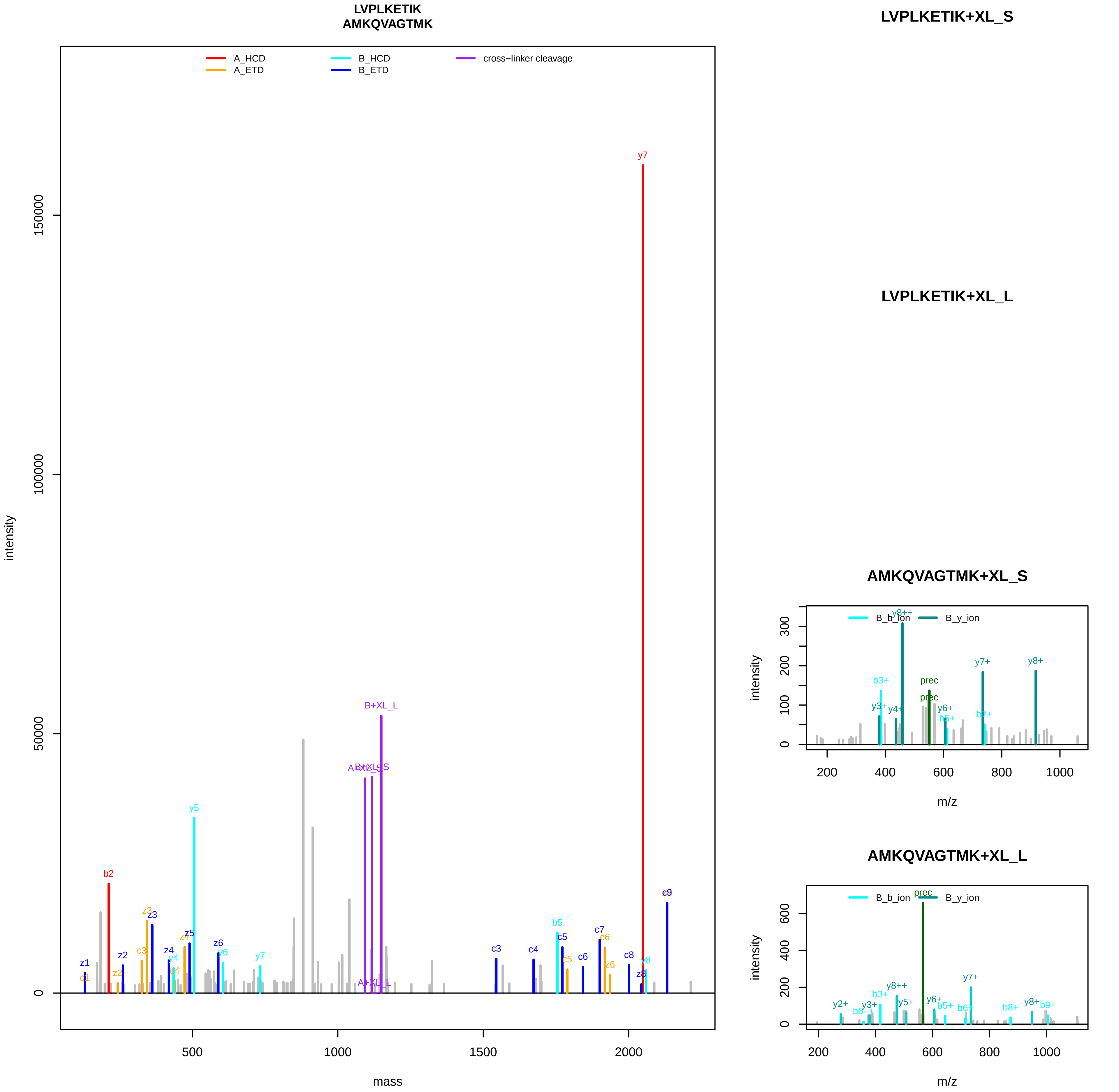
LVPLKETIK
AMKQVAGTMK
A_HCD
A_ETD
B_HCD
B_ETD
cross−linker cleavage
intensity
mass
0
50000
100000
150000
500
1000
1500
2000
y7
b2
y5
B+XL_L
A+XL_S
B+XL_S
A+XL_L
c9
LVPLKETIK+XL_S
LVPLKETIK+XL_L
AMKQVAGTMK+XL_S
AMKQVAGTMK+XL_L
B_b_ion
B_y_ion
m/z
prec
y8++
y7+
y8+
b3+
y3+
y4+
y6+
b5+
b7+
y2+
b6++
y5+
b6+
b8+
b9+
